# Reduced chemically modified graphene oxide for supercapacitor electrode

**DOI:** 10.1186/1556-276X-9-535

**Published:** 2014-09-29

**Authors:** Balasubramaniyan Rajagopalan, Jin Suk Chung

**Affiliations:** 1 School of Chemical Engineering, University of Ulsan, 93 Daehakro, Namgu, Ulsan 680-749, Republic of Korea

**Keywords:** Graphene oxide, Chemical reduction, Active material, Supercapacitor

## Abstract

An efficient active material for supercapacitor electrodes is prepared by reacting potassium hydroxide (KOH) with graphene oxide followed by chemical reduction with hydrazine. The electrochemical performance of KOH treated graphene oxide reduced for 24 h (reduced chemically modified graphene oxide, RCMGO-24) exhibits a specific capacitance of 253 F g^-1^ at 0.2 A g^-1^ in 2 M H_2_SO_4_ compared to a value of 141 F g^-1^ for graphene oxide reduced for 24 h (RGO-24), and good cyclic stability up to 3,000 cycles. Interestingly, RCMGO-24 demonstrated a higher specific capacitance and excellent cycle stability due to its residual oxygen functional groups that accelerate the faradaic reactions and aid in faster wetting. This non-annealed strategy offers the potential for simple and cost-effective preparation of an active material for a supercapacitor electrode.

## Background

Supercapacitors or electrochemical capacitors, powerful energy storage systems used for a wide range of energy capture and storage applications, provide clean energy with almost zero waste emission [1-3]. However, there is a growing demand for supercapacitors with higher energy density. To meet this demand, advanced electrode materials, specific electrolytes, and tuning the electrode/electrolyte interface properties for high capacitance and a longer life cycle are necessary [4,5]. Recently, graphene [6], carbon nanotubes [7], metal oxides [8], and conducting polymers [9] have been extensively studied for use as electrodes in supercapacitors. Among these, two-dimensional (2D) graphene has attracted a great attention due to its distinct high surface area, electrical conductivity, and mechanical stability [3,10]. In recent times, chemically reduced graphene [5,11], activated graphene [12,13], and graphene nanocomposites [14,15] have been broadly studied for use as electrodes in supercapacitors.

Graphene can be prepared by the chemical reduction of graphene oxide (GO) [16-18], epitaxial growth on metal surfaces [19], or mechanical exfoliation [20]. The chemical reduction of GO has been frequently applied for large-scale production of graphene sheets. The electrical conductivity and dispersibility of graphene is probably due to chemical reduction [16,17,21]. The electrochemical performance of an electrode material deeply depends on the electrical conductivity, surface area, wetting of the electrode, and permeability of the electrolyte solution [5,11,22]. The permeability of an electrolyte solution can be increased considerably due to its porous nature or faster wetting induced by the residual functional groups of the graphene sheets [5]. Active spaces and pores present in the graphene sheets transport the electrolyte ions efficiently between the interfaces and improve the specific capacitance [22]. The aggregated graphene sheets of thermally annealed graphene perform like graphite and decrease the effective electrolyte diffusion. However, the presence of hydrophilic functional groups on the graphene sheets efficiently improves the wetting between graphene and polar electrolytes, and also inhibits the aggregation of graphene sheets. Additionally, this oxygen functional group has a pseudocapacitance effect with the double-layer capacitor [23]. A few solution-based approaches have been reported for applying graphene in supercapacitors such as reduction of surfactant-stabilized GO [5], solvothermally reduced graphene [23], poly(ionic liquid)-modified graphene [24], and partially reduced GO [11]. To enhance the energy storage capacitance of graphene, the introduction of the pseudocapacitance property is essential. The pseudocapacitance property supports a synergistic effect on both double-layer capacitance and pseudocapacitance [24,25]. In fact, the presence of the residual oxygen groups on the graphene sheets induces the pseudocapacitive effects (electrochemical reduction and oxidation) and promotes good wettability of the electrodes [5,11,26]. To increase the energy storage capacitance of the reduced graphene oxide (RGO), they must have some residual oxygen functional groups for pseudocapacitance and faster electrolyte permeability.

Herein we report on the preparation, through simple reactions of KOH with graphene oxide followed by hydrazine reduction, of an active material of graphene with adequate dispersibility and high storage capacity. The reactions of KOH induce the formation of oxygen functional groups and defects on graphene sheets. The reduced KOH-treated graphene oxide was analyzed with thermal gravimetric analysis (TGA), Raman, Fourier transform infrared spectroscopy (FT-IR), and electrochemical charge/discharge cycles to elucidate the physical and electrochemical performances. KOH-treated reduced graphene oxide exhibits adequate dispersibility in an aqueous medium; consequently, it induces good wettability in an aqueous electrolyte solution. Due to the presence of residual oxygen functional groups for pseudocapacitance in this KOH-treated graphene oxide, higher storage capacitance and good electrochemical stability is achieved.

## Methods

### Materials

Expandable graphite (Grade 1721, Asbury, NJ, USA) was kindly provided by Asbury Carbon. Concentrated sulfuric acid (H_2_SO_4_), potassium permanganate (KMnO_4_), hydrochloric acid (HCl), and hydrogen peroxide (H_2_O_2_) were purchased from Samchun Chemical (Gyeonggi-do, Korea). Hydrazine monohydrate (N_2_H_4_∙H_2_O) and KOH was purchased from Sigma-Aldrich (St. Louis, MO, USA). All chemicals were used as received without further purification.

### Preparation of chemically modified GO

GO was prepared from expandable graphite according to the modified Hummers method [17]. To prepare the chemically modified GO (CMGO), 200 mg of GO was dispersed in 100 mL of deionized water, stirred and sonicated in an ultrasonic bath for 30 min. Approximately 1 g of KOH was added to the GO suspension and stirred for 6 h, while the color of the suspension changed from yellow to black. The suspension was then centrifuged at 8,000 rpm. The precipitate was redispersed in deionized water and 1 g of KOH was added while stirring. This procedure was repeated three times. Finally, the GO was completely functionalized with KOH, becoming CMGO, as the color of the suspension was completely changed to dark black.

### Chemical reduction of GO and CMGO

The GO was dispersed in deionized water, placed in an oil bath and the temperature was set at 95°C. Hydrazine monohydrate (1:5, GO/hydrazine) was added and stirred for each reaction time. The reduced GO was filtered and washed with water, then dried in an oven at 80°C for 24 h. The reduced CMGO (RCMGO) sample was also prepared by applying same reaction conditions of RGO to CMGO. The sample of GO reduced for 6 h, 24 h without KOH treatment, and CMGO reduced for 24 h were named RGO-6, RGO-24, and RCMGO-24, respectively.

### Electrode preparation and electrochemical measurements

All electrochemical measurements were conducted in a three-electrode cell assembly equipped with a reference electrode (Ag/AgCl), platinum wire as a counter electrode, and a glassy carbon working electrode. To fabricate the electrode, 85% active material, 10% super P, and 5% polytetrafluoroethylene were mixed with ethanol and then dried to form a paste. Electrochemical performance was measured in a 2 M aqueous sulfuric acid solution as an electrolyte. The specific capacitances of RGO-6, RGO-24, and RCMGO-24 were measured at 0.2, 0.5, and 1 A g^-1^. Cyclic voltammetry (CV) was conducted at 10 mV s^-1^ and electrochemical impedance spectra (EIS) was measured at frequencies from 100 kHz to 0.01 Hz.

### Instruments

Morphologies of the RGO-24 and RCMGO-24 were characterized via field emission scanning electron microscopy (FE-SEM, JSM-6500 F, JEOL, Akishima-shi, Japan). Thermal properties were characterized using thermal gravimetric analysis (TGA, Q50, TA, New Castle, DE, USA). The electrical conductivity of the graphene pellet was determined by the four-point probe method (CMT-10 MP, AIT). The functional groups of the RGO were determined by FT-IR on a Nicolet IR 200 FT-IR spectrometer (Thermo Scientific, Waltham, MA, USA) in transmission mode, and Raman (Thermo scientific, DXR) with 633-nm wavelength incident laser light was used to characterize the degree of reduction on the graphene sheets. X-ray diffraction (XRD) analyses were performed on a high-power X-ray diffractometer (Rigaku, Shibuya-ku, Japan) at 2*θ* range from 2° to 40°. X-ray photoelectron spectroscopy (XPS) was taken on a Thermo Fisher instrument using Al Kα radiation (energy range 200 to 3 keV). Galvanostatic charge/discharge and CV were characterized using a Won A Tech (WBCS 3000) battery tester. EIS was measured using BioLogic Science Instruments (Claix, France).

## Results and discussion

**Figure 1:**
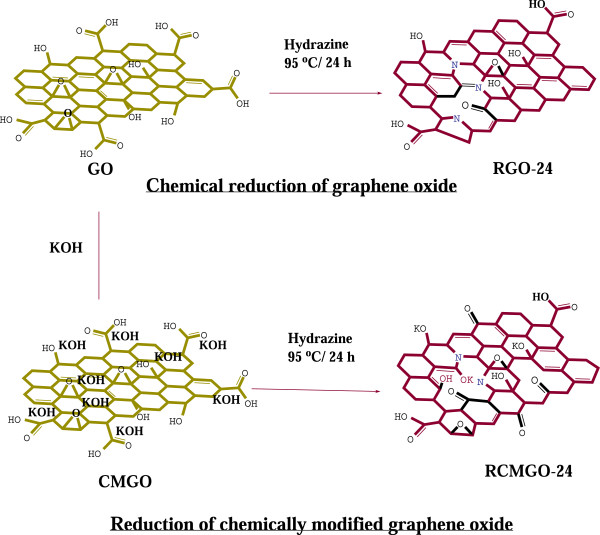
**Preparation method of graphene samples.** Diagrammatic representation for the preparation of RGO-24 and RCMGO-24.

**Figure 2:**
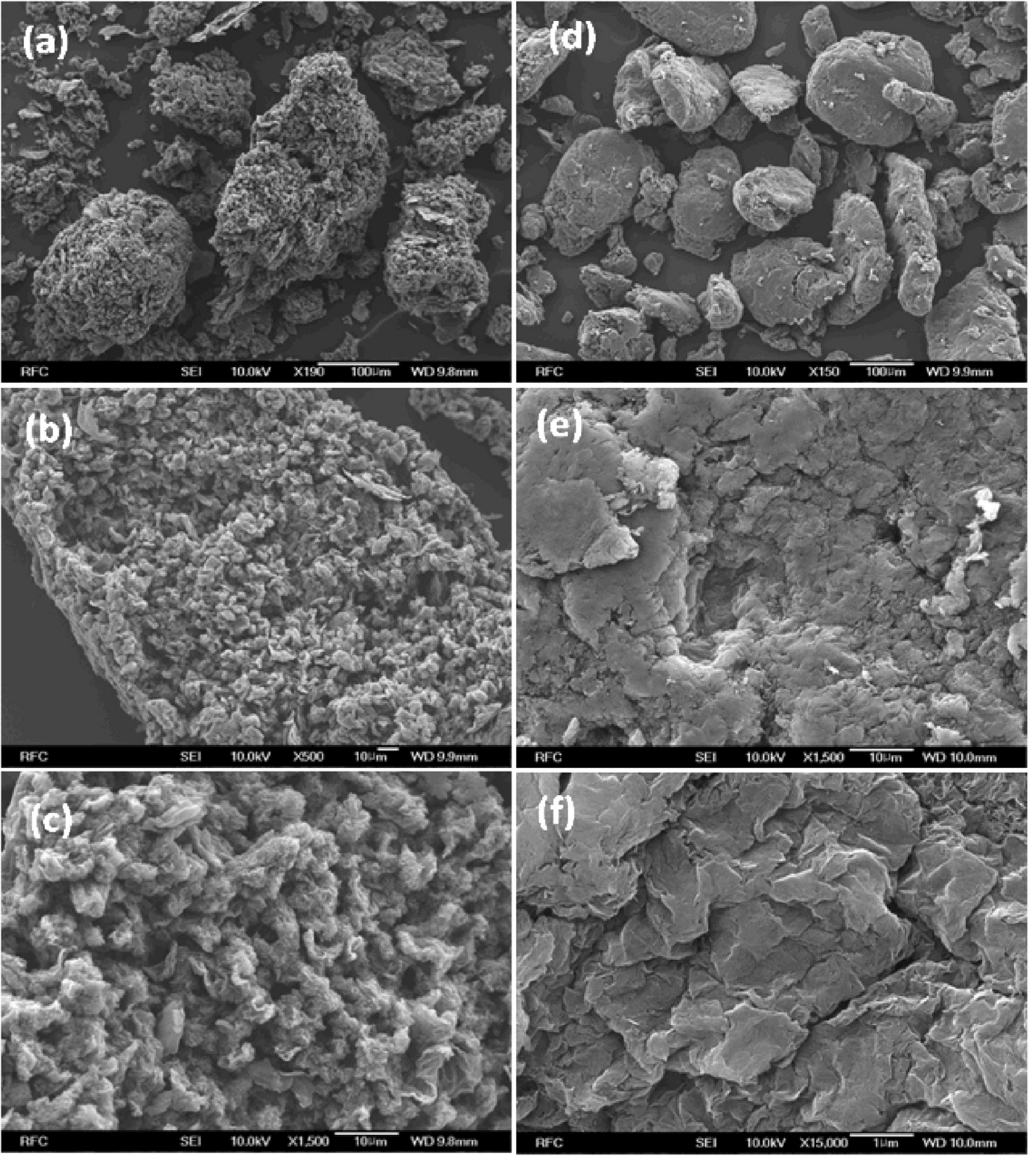
**FE-SEM images of graphene samples.** FE-SEM images of **(a-c)** RCMGO-24 and **(d-f)** RGO-24 at different magnifications.

As shown in Figure 1, the reactions of KOH with GO extensively decorated the K ions on the GO sheets through reactions involving oxygen groups such as hydroxyl, epoxy, and carboxyl groups [27]. Throughout the chemical reactions, KOH controlled the complete reduction by maintaining the residual oxygen functional groups. Meanwhile, the K ions were intercalated or weakly bound on the hydroxyl and epoxy groups of GO [27]. The surface morphology of graphene was analyzed using FE-SEM. Figure 2a-f shows the FE-SEM images of RGO-24 and RCMGO-24 at different magnifications. The FE-SEM images of RGO-24 showed the restacked and aggregated arrangement of graphene sheets. The restacking of RGO-24 was mostly due to the loss of repulsion between graphene sheets caused by hydrazine reduction. In the case of using KOH treatment, the weakly bound K ions protected the epoxy and hydroxyl groups from removal by hydrazine and effectively inhibited restacking (Figure 2a-c). For energy storage applications, electrodes with high active spaces are essential for efficient electrolyte diffusion. In RCMGO-24, the presence of enough active spaces between the crumpled graphene sheets may be capable of inducing the electrolyte efficiently between the electrodes [25]. Recently, higher performance in the supercapacitor has been observed at the crumpled graphene sheets due to the faster electrolyte diffusion rate inside the crumpled spaces [25].

**Figure 3:**
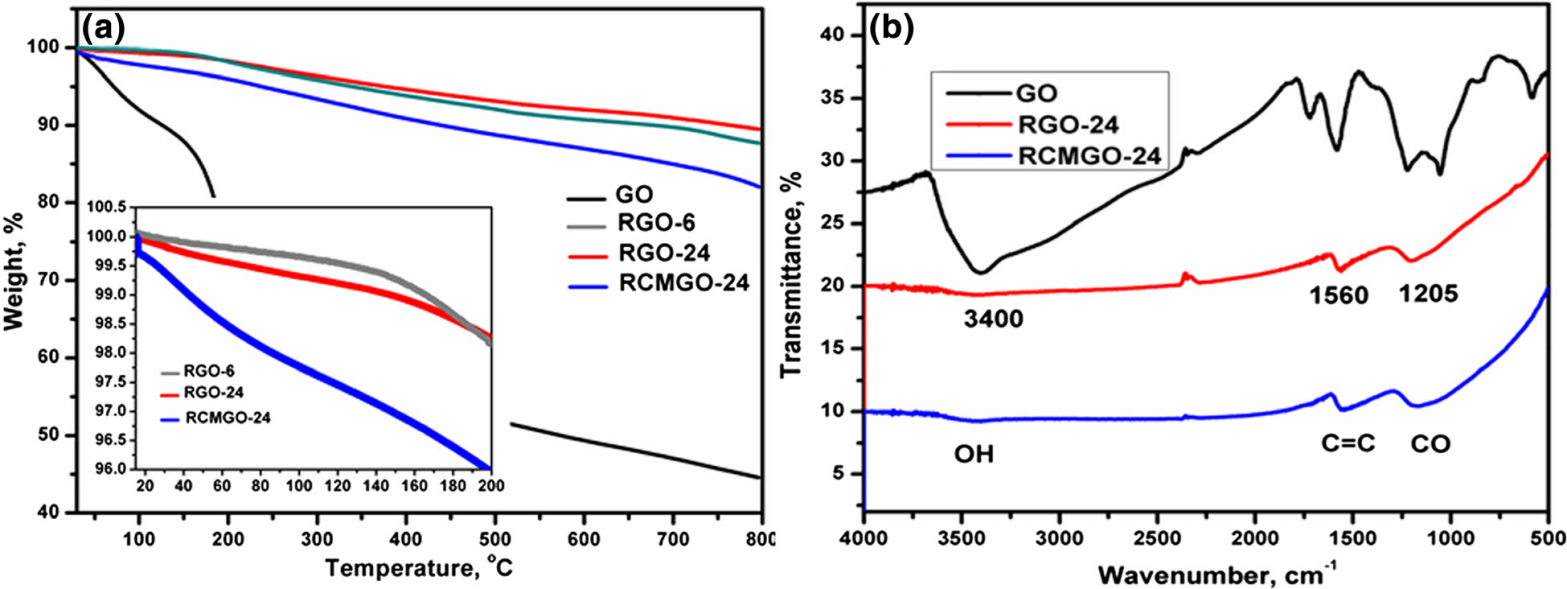
**TGA and FT-IR spectra of graphene samples. (a)** TGA of GO, RGO-6, RGO-24, and RCMGO-24 measured under N atmosphere, **(b)** FT-IR spectra of GO, RGO-24, and RCMGO-24.

TGA was carried out to observe the presence of functional groups on the graphene sheets. As shown in Figure 3a, the initial weight loss of GO from 100°C to 200°C was attributed to the decomposition of residual water and oxygen. The gradual weight loss observed from 200°C to 650°C might have resulted from the decomposition of carboxyl, hydroxyl, and epoxy groups at the edge and basal planes, respectively. Interestingly, RGO-6 and RGO-24 exhibited almost similar thermal stability up to 800°C, inferred that most of the functional groups were rapidly removed by the use of chemical reduction. The small weight loss up to 200°C for RGO-24 in comparison to RGO-6 (Figure 3a) is due to the degradation of labile oxygen in the defect areas, which was formed with respect to increasing reaction time. The thermal stability of RCMGO-24 is comparable to RGO-6 and RGO-24. It was observed that the reactions of KOH potentially reduce the degree of reduction, while also creating new oxygen functional groups; thus, it was gradually removed upon heating to 200°C [28]. These residual functional groups promote the dispersibility of RCMGO-24 in an aqueous medium or ethanol up to 0.3 mg mL^-1^. The electrical conductivity of RGO-24 was measured to be as high as 1,980 S m^-1^. On the other hand, the residual functional groups obtained from KOH reaction diminish electron transport of RCMGO-24 and thus the electrical conductivity decreases to 431 S m^-1^. Nevertheless, there is enough electrical conductivity for charge transport during charge/discharge cycles.

As shown in FT-IR spectra (Figure 3b) the broad peak at 3,410 cm^-1^ originates from the stretching vibration of OH groups, and a peak at 1,727 cm^-1^ is due to the C = O stretching vibrations of carbonyl and carboxyl groups. The C = C of an un-oxidized graphitic domain gives a skeletal vibration at 1,590 cm^-1^. The peaks at 1,230 and 1,050 cm^-1^ are characteristics of carboxyl C-O deformation and alkoxy C-O stretching vibrations, respectively. As seen in the figure, the peak intensities of RGO-24 and RCMGO-24 were lower than those of GO, suggesting effective removal of functional groups by hydrazine. However, these graphene samples still have some surface functional groups over the basal and edge planes of the graphene sheets, which were observed at 3,400, 1,560, and 1,205 cm^-1^ for residual -OH, C = C, and C-O groups, respectively. The broad peak observed at 1,205 cm^-1^ for RCMGO-24 denotes the greater presence of residual oxygen functional groups.

**Figure 4:**
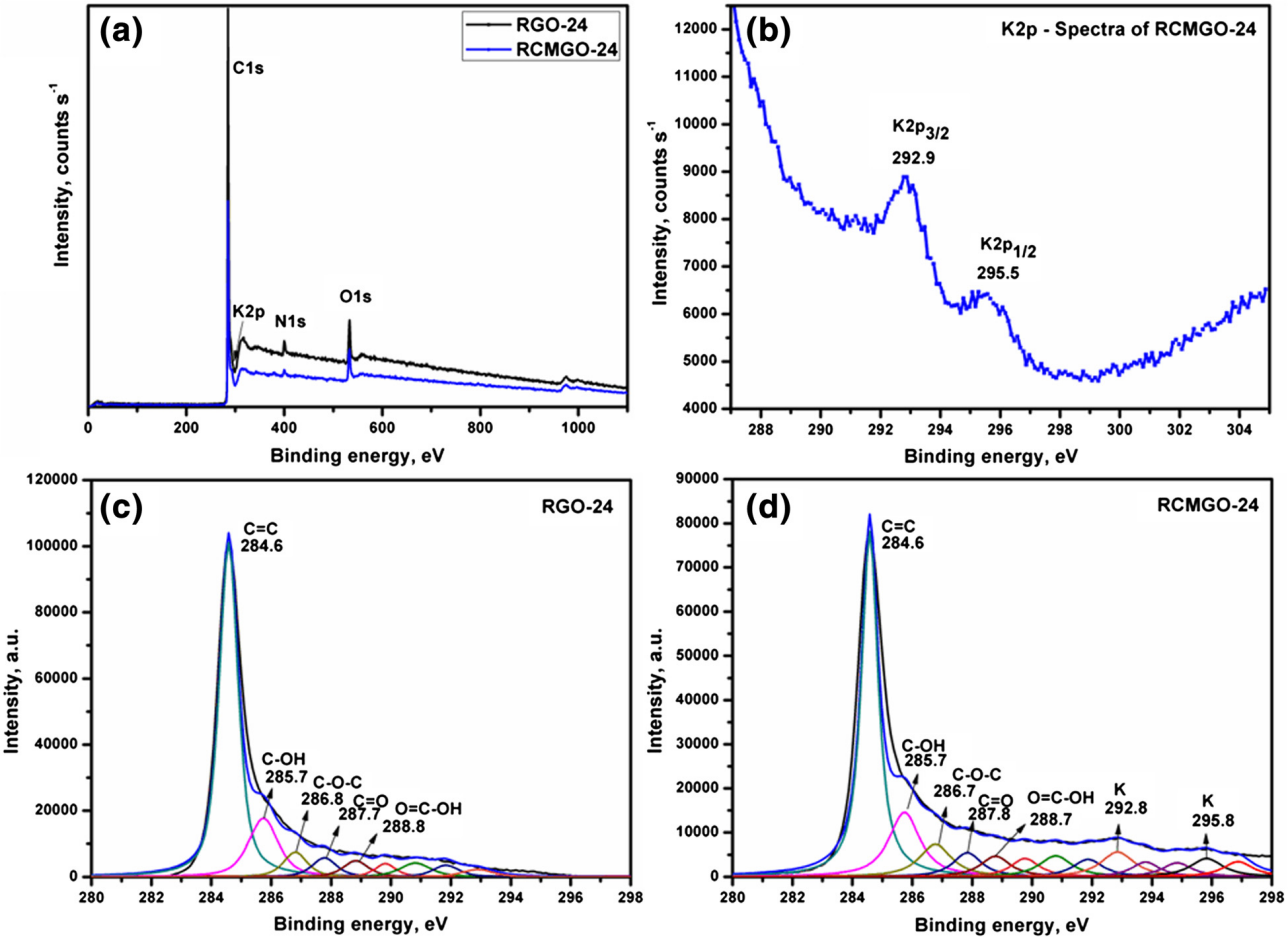
**XPS analysis of graphene samples.** XPS general spectra of RGO-24 and RCMGO-24 **(a)**, determination of K from K2p spectra of RCMGO-24 **(b)**, CIs XPS spectra of RGO-24 **(c)**, and C1s XPS spectra of RCMGO-24 **(d)**.

The presence of C, N, O, and K elements in the graphene sheets was confirmed by XPS spectra for both RGO-24 and RCMGO-24 (Figure 4a). The existence of a significant amount of K in RCMGO-24 was identified by the formation of K2p_3/2_ and K2p_1/2_ peaks at 292.9 and 295.5 eV, respectively (Figure 4b). The K content was determined to be 0.14% (Table 1), which was intercalated or weakly bound to the graphene sheets. It should be noted that, the K ions on the graphene sheet is in trace amounts and it can be washed out. Figure 4c,d shows the C1s spectra of RGO-24 and RCMGO-24. Both RCMGO-24 and RGO-24 demonstrate an intense C = C peak indicating greater reduction of GO. The reduction of GO was further supported by the weight percentage (wt.%) of atomic C: maximum values of 91.33% and 89.34% were determined for RGO-24 and RCMGO-24, respectively. The presence of residual functional groups such as hydroxyl, epoxy, and COOH groups were found in both RGO-24 and RCMGO-24. The elemental analysis presents 6.47 and 8.81% of oxygen at RGO-24 and RCMGO-24, respectively. The C1s spectra of RCMGO-24 showed several small peaks up to 298 eV, indicating the formation of unidentified functional groups on the graphene sheets due to the KOH reaction. Note that no other peaks, except those corresponding to -OH, C-O, and C = C were observed in the FT-IR spectra. Therefore, the small domains obtained may be due to the residual oxygen groups bound with carbon attained through the KOH reactions. The elemental analyses of RCMGO-24 and RGO-24 show a significant amount of N doping on graphene sheets. Hydrazine acts as a reducing agent, as well as a good N dopant to graphene sheets; the maximum level of N was found to be 2.2% and 1.7% with RGO-24 and RCMGO-24, respectively (Table 1). This residual presence of N doping suggests that both RCMGO-24 and RGO-24 experience defects in the 2D layer [25].

**Table 1:** XPS elemental analysis of RGO-24 and RCMGO-24

**Samples**	**Elemental composition, %**					
	**C**	**O**	**N**	**K**	**C/O**	**C/N**
RGO-24	91.33	6.47	2.2	0	14.11	41.5
RCMGO-24	89.34	8.81	1.71	0.14	10.13	52.2

**Figure 5:**
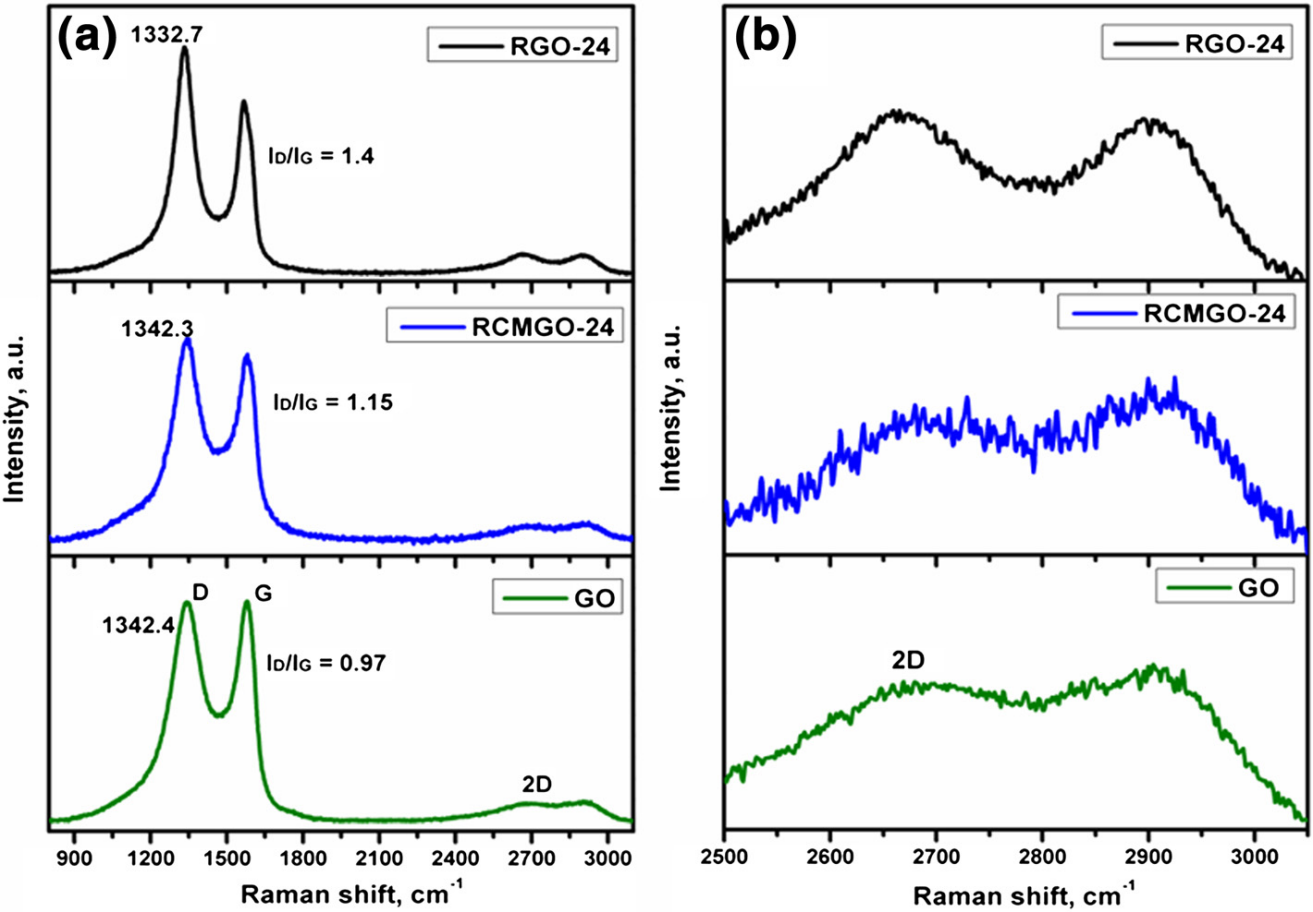
**Raman spectroscopy of graphene samples. (a)** General Raman spectra, **(b)** evaluation of 2D peak of GO, RGO-24, and RCMGO-24 taken on a laser power of 633 nm.

**Figure 6:**
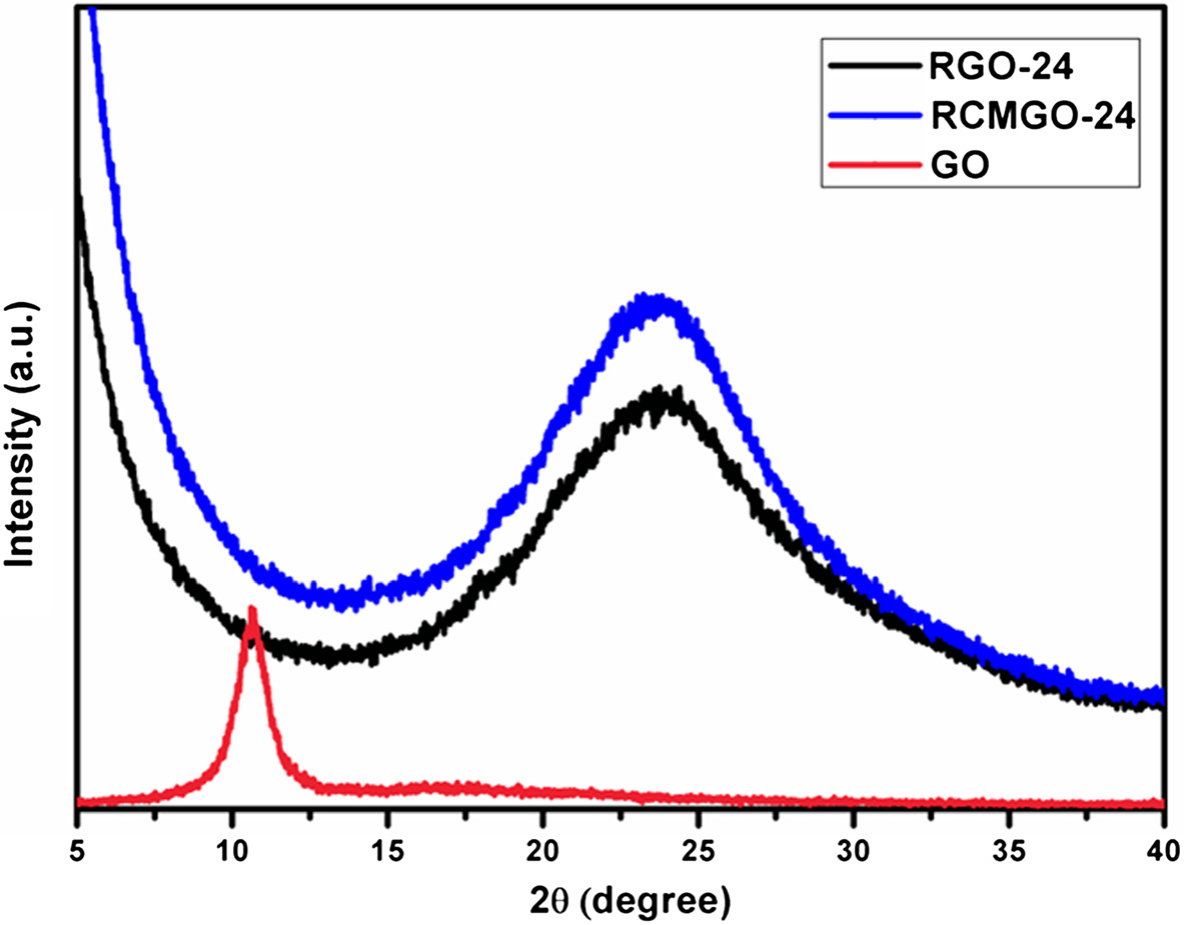
**XRD analysis of graphene samples.** XRD patterns of GO, RGO-24, and RCMGO-24 measured at 2*θ* range from 2° to 40°.

Raman spectroscopy was used to characterize the structures of graphene by comparing G and D bands. The G band corresponds to the E^2g^ mode with characteristics of sp^2^ bonding, while the D band is attributed to the defects and disordered nature of graphene sheets. Figure 5a shows the comparison of Raman spectra for GO, RCMGO-24, and RGO-24. The Raman spectrum of GO shows two absorption bands at 1,342.4 and 1,579 cm^-1^ corresponding to D and G bands, respectively. During reduction, the characteristic D band of GO shifted to a lower number [25]. For RGO-24, the D band of GO shifted from 1,342.4 to 1,332.7 cm^-1^ indicating an extreme reduction of GO. Meanwhile, RCMGO-24 (1,342.3 cm^-1^) yielded a value very near to that of GO. Interestingly, the reactions of CMGO at higher temperature accelerated the formation of new oxygen functional groups, so that the D band of RCMGO-24 was observed at a similar level to that of GO. Moreover, the degree of reduction and defects were also determined by calculating the *I*_D_/*I*_G_ ratio of GO, RCMGO-24, and RGO-24. The *I*_D_/*I*_G_ ratio of GO was calculated to be 0.97. After the reduction, the D band of GO increased to more than that of the G band (*I*_D_/*I*_G_ = 1.4), suggesting that the reaction of hydrazine formed a higher reduction and larger number of defects over the graphene sheets [11,29]. It is interesting to note that the *I*_D_/*I*_G_ ratio of RCMGO-24 is much smaller than that of RGO-24. The *I*_D_/*I*_G_ ratio of RCMGO-24 was calculated to be 1.15; this small number results from the formation of a large number of oxygen functional groups on the defected areas. From XRD analysis (Figure 6), compared with RGO-24, the 002 peaks of RCMGO-24 show a significantly increased intensity and dramatically broadened width, suggesting that RCMGO-24 experiences crumpling and little stacking [25]. The multilayer stacking of graphene sheets can also be confirmed by evaluating the characteristic 2D band from Raman spectroscopy (Figure 5b). The intensity of the 2D band was higher for RGO-24 than for RCMGO-24, indicating a multilayer assembly of graphene sheets [25]. The reactions of KOH potentially decrease the layer stacking of graphene sheets; this is further supported by the FE-SEM images of RGO-24 and RCMGO-24. In contrast, the RCMGO-24 formed stacked-crumpled graphene sheets and fully reduced RGO-24 (*I*_D_/*I*_G_ = 1.4) restored its graphitic structure, and then formed a stacked, multilayer graphene.

**Figure 7:**
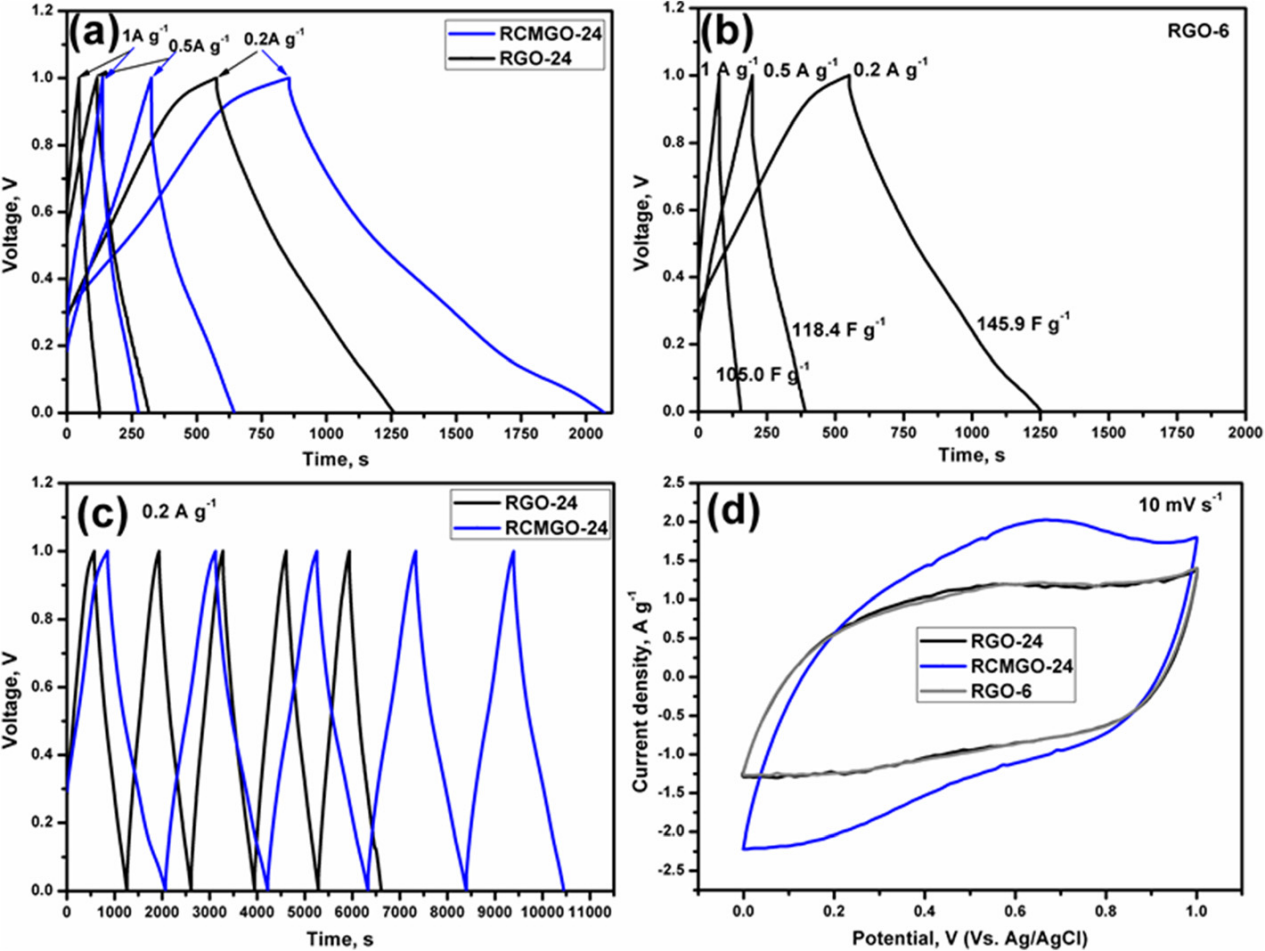
**Electrochemical performances of graphene samples.** Galvanostatic charge/discharge cycles of **(a)** RCMGO-24 and RGO-24, **(b)** RGO-6 at 0.2, 0.5, and 1A g^-1^. **(c)** Galvanostatic charge/discharge cycles of RCMGO-24 and RGO-24 at 0.2 A g^-1^ for 5 cycles. **(d)** CV of RGO-6, RGO-24, and RCMGO-24 at a scan rate of 10 mV s^-1^.

Next, we performed the analysis in a supercapacitor electrode to evaluate the storage capacitance and cyclic stability of RGO-24 and RCMGO-24. We believe that the crumpled structure and residual oxygen functional groups obtained from KOH treatment can effectively increase the specific capacitance of graphene sheets. Galvanostatic charge/discharge was conducted at current densities of 0.2, 0.5, and 1 A g^-1^ under a potential window of 0 to 1 V. The slope of a full discharge curve (except internal resistance (IR) drop) was chosen to measure the specific capacitance of RGO-24 and RCMGO-24. The specific capacitance of graphene was determined by using the following equation:

$$C_{\text{spec}}=\frac{I}{m\;\times \;\left(\mathrm{dv}/\mathrm{dt}\right)}$$

where *C*_spec_ is the specific capacitance of an electrode, *I* is the current density (A), *m* is the mass of the active material (g), and *dv/dt* is the slope of the discharge curve. Figure 7a depicts the comparison of first charge/discharge cycles of RGO-24 and RCMGO-24 at current densities of 0.2, 0.5, and 1 A g^-1^ measured after the 10 CV (10 mV s^-1^) cycles. The discharge curves are close to linear over the whole potential range, implying perfect capacitance behavior [30]. Secondly, the IR drop of RGO-24 and RCMGO-24 was very small due to the very low equivalent series resistance (ESR) of the supercapacitor electrodes. Low IR is vital for energy-storing devices, as it increases the charge transfer reaction during the charging/discharging processes. At increasing current densities, the discharge cycles of RGO-24 were shortened in comparison to RCMGO-24. Conversely, the discharge cycles of RCMGO-24 demonstrated a linear deviation from 0.6 to 0 V. Table 2 shows the specific capacitance of RGO-24 and RCMGO-24 at 0.2, 0.5, and 1A g^-1^. The maximum specific capacitances of RGO-24 and RCMGO-24 were observed at 141 and 253 F g^-1^at 0.2 A g^-1^, respectively. The specific capacitance of RCMGO-24 is comparable to the value reported for reduced graphene supercapacitors [5,23,24]. The specific capacitance decreases at increasing current densities. The specific capacitance of RCMGO-24 at each applied current density was significantly higher than that of RGO-24 (Table 2). As shown in Figure 7b, the specific capacitance of RGO-6 was found to be increased, but only slightly in comparison to RGO-24. The obtained excellent specific capacitance of RCMGO-24 was attributed to the contribution of pseudocapacitance with the electrical double-layer capacitor (EDLC). Further, the pseudocapacitance was confirmed by comparing the discharge slopes of RCMGO-24 and RGO-24. We particularly compare the physical and electrochemical properties of RCMGO-24 and RGO-24 throughout this article due to the same reaction conditions applied to both samples.

**Table 2:** Comparison of the specific capacitance of RGO-24 and RCMGO-24

**Samples**	**Specific capacitance, F g**^**-1**^		
	**0.2 A g**^**-1**^	**0.5 A g**^**-1**^	**1 A g**^**-1**^
RCMGO-24	253	190	157
RGO-24	141	109	84.7

The discharge cycles of RCMGO-24 and RGO-24 at 0.2 A g^-1^ (Figure 7c) clearly shows there is a deviation of linearity in RCMGO-24 from 0.6 to 0.0 V. The specific capacitance measured from 0.6 to 0.0 V is found to be much higher than that of 0.6 to 1.0 V (Table 3). These interesting results represent contributions from the EDLC (0.6 to 1 V) and pseudocapacitance (0.6 to 0.0 V) [23]. A linear discharge curve results from the double layer capacitors [3,22]. However, the linear discharge curves observed in RGO-24 gave only small numbers, which were attributed to the lower pseudocapacitance behavior. Moreover, the CV (Figure 7d) of RGO-6 and RGO-24 gave a rectangular cycles at 10 mV s^-1^, confirming the domination of EDLC characteristics. However, RCMGO-24 demonstrated an elliptical cycle with a higher storage area. Based on these characteristic discharge plateaus and the CV results of RCMGO-24, the contributions of both the pseudocapacitance and EDLC can be inferred. It is believed that the observed pseudocapacitance resulted from the oxygen containing functional groups and H^+^ ions [23]. Thus, the proton-rich sulfuric acid is the preferred electrolyte solution. Only carbonyl and hydroxyl functional groups offered a good pseudocapacitance effect compared with the other oxygen functional groups of graphene. As discussed, the oxygen obtained from the KOH reaction and the residual oxygen groups only play a major role during the pseudocapacitance effect. RGO-24 also experienced the pseudocapacitance effect (Table 3), but it was very low in comparison to RCMGO-24 due to the lack of oxygen groups. We tried to reduce the reaction time of chemical reduction (RGO-6) for preventing the complete reduction. However, the chemical reduction inevitably removed the most of the functional groups in a short period. It is believed that, the oxygen functional groups were formed on the defected areas (Figure 1), defect increased based on the reaction time, and it is very clear from the TGA report (Figure 3a). The defect and residual functional groups were increases in the order of RGO-6, RGO-24 to RCMGO-24. It is inferred that KOH reaction potentially increases the defect sites, and produced the oxygen functional groups in the respected areas. The electrical conductivity did not meaningfully contribute to the specific capacitance of the electrodes, because the capacitance is directly related to the rapid electrolyte accessibility and residual oxygen groups of the graphene. Even though the electrical conductivity of RGO-24 is much larger than RCMGO-24, the lack of oxygen functional groups for pseudocapacitance decreases the performance of RGO-24.

**Table 3:** Comparison of the pseudocapacitance effect of RGO-24 and RCMGO-24

**Sample name**	**Voltage, V**	**Specific capacitance (F g**^**-1**^**) at 0.2 A g**^**-1**^				
		**1st**	**2nd**	**3rd**	**4th**	**5th**
RCMGO-24	(i) 0.0 to 0.6	323.7	286	279	275	276
	(ii) 0.6 to 1.0	123.4	129	118	118	114
RGO-24	(i) 0.0 to 0.6	170	166	166.6	168.2	172.5
	(ii) 0.6 to 1.0	88.1	87.3	86.9	86.9	75.7

**Figure 8:**
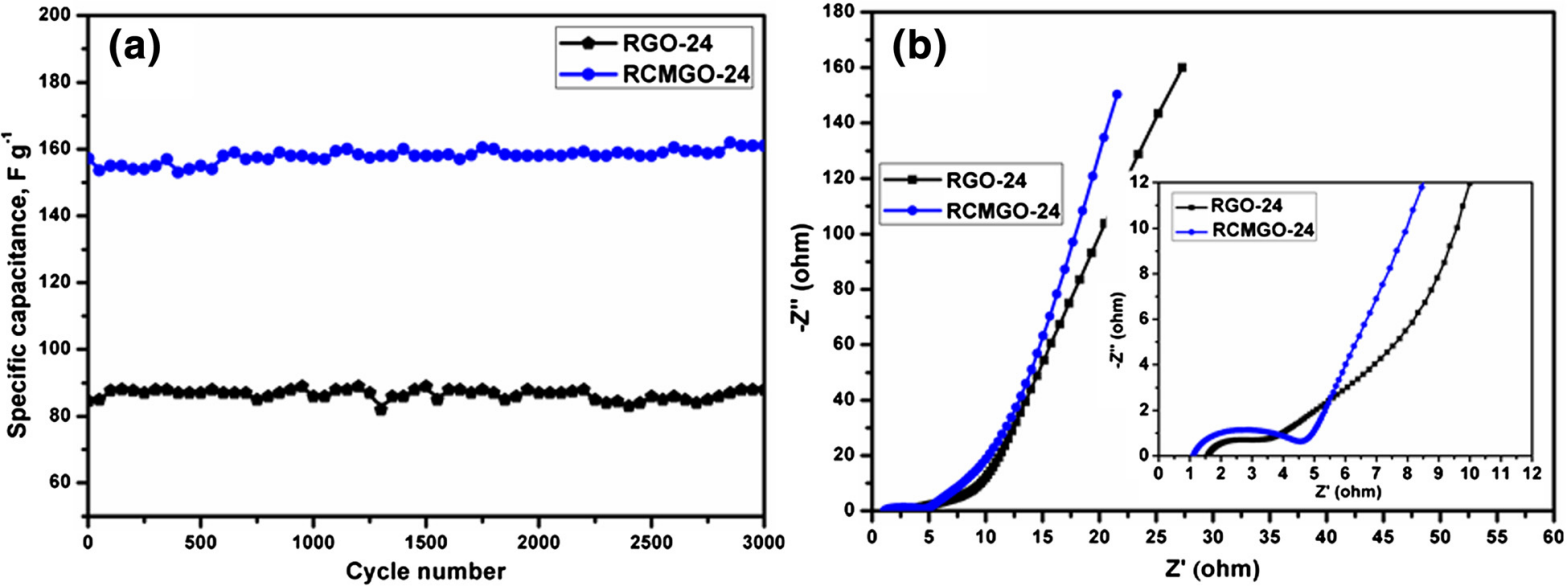
**Cyclic test and EIS analysis. (a)** The cyclic stability of RCMGO-24 and RGO-24 at 1A g^-1^ for 3,000 cycles. **(b)** EIS of RCMGO-24 and RGO-24 at the frequency range from 100 kHz to 0.01 Hz.

The cyclic stabilities of RCMGO-24 and RGO-24 were evaluated by repeating the charge/discharge for 3,000 cycles at 1 A g^-1^ (Figure 8a). RGO-24 and RCMGO-24 retained the initial capacitance of 103.8% and 102.5%, respectively, at the 3,000th cycle, demonstrating the excellent electrochemical stability. Meanwhile, a much higher specific capacitance of RCMGO-24 revealed a large number of functional groups involved in the pseudocapacitance effect.

Figure 8b shows the Nyquist plots of RGO-24 and RCMGO-24 electrodes. It is well-known that the semicircle in the medium frequency region is ascribed to the charge transfer process at the electrode-electrolyte interface, while the straight line at the low frequency region is related to the diffusion limitation of lithium ions [3,31]. An intercept of the semicircle at the *x*-axis (starting point) represents ESR and it corresponds to the internal charge, charge/discharge rate, and wettability of the electrode, [4,32,33]. The EIS of RGO-24 demonstrated a small semicircle and slight vertical line. However, RCMGO-24 is too broad and more vertical at the low frequency region. The observed broader semicircle of RCMGO-24 reflects the resistance to charge transfer reactions by the presence of labile oxygen groups. The angle of the Warburg region is higher for RCMGO-24 due to a faster electrolyte diffusion on the electrode-electrolyte interfaces. Moreover, the ESR shows a lower resistance value for RCMGO-24 indicating a lower internal resistance, good charge/discharge rate, and higher wettability of the electrode. The straight line of RCMGO-24 at the low frequency region approaches the *y*-axis in comparison to RGO-24, thereby confirming the contribution of higher EDLC characteristics. These interesting characteristics of RCMGO-24 indicate that the KOH treatment decreased electrical conductivity, but it significantly increased the electrode wetting, electrolyte diffusion, EDLC, and more pseudocapacitance in comparison to RGO-24.

A non-annealing strategy of KOH-treated GO was developed for preparing RCMGO-24 containing residual functional groups. The reactions of KOH efficiently created the oxygen functional groups and were involved in the pseudocapacitance effect; the same characteristic features were observed using a solvothermal method at elevated temperature (150°C) [23]. The crumpled, N-doped, and residual oxygen functionalized RCMGO-24 endowed a faster electrolyte diffusion rate and more pseudocapacitance with EDLC, resulting in an improved electrochemical performance as compared with RGO-24. Therefore, this non-annealed strategy to prepare RGO is simple, cost effective, and a potentially effective method for high-performance supercapacitor electrodes.

## Conclusions

We have investigated the effect of KOH treatment of GO on supercapacitor performance at various current densities. The Raman, TGA, FT-IR, and XPS spectra of RCMGO-24 confirmed that KOH functionalization efficiently prevents complete reduction and supplies residual functional groups on graphene sheets. The excellent performance of supercapacitor electrodes obtained with RCMGO-24 illustrated the key role of the KOH treatment. It promoted faster wetting of the electrode, more pseudocapacitance, and the permeation of an electrolyte solution. The electrochemical stability of RCMGO-24 was sufficient and maintained capacitance retention for up to 3,000 cycles, demonstrating that it is an excellent active material for supercapacitor electrodes.

## Abbreviations

CV: cyclic voltammetry; EIS: electrochemical impedance spectra; ESR: equivalent series resistance; EDLC: electrical double-layer capacitor; FT-IR: Fourier transform infrared spectroscopy; GO: graphene oxide; IR: internal resistance; RGO: reduced graphene oxide; RCMGO: reduced chemically modified graphene oxide; FE-SEM: field emission scanning electron microscopy; TGA: thermal gravimetric analysis; XRD: X-ray diffraction; XPS: X-ray photoelectron spectroscopy.

## Competing interests

The authors declare that they have no competing interests.

## Authors’ contributions

BR designed the work, carried out the experiment, and finalized the manuscript. JSC supervised the work and revised the manuscript. All authors read and approved the final manuscript.

## Authors’ information

BR is a PhD student at the University of Ulsan (UOU). JSC has a PhD degree and is a professor at UOU.
